# Hepatic manifestations of ciliopathies: genetic causes and clinical update

**DOI:** 10.1007/s00431-026-07468-3

**Published:** 2026-10-09

**Authors:** Robert Hegarty, Yoh Zen, John A. Sayer

**Affiliations:** 1 Paediatric Liver, GI and Nutrition Centre, King’s College Hospital NHS Foundation Trust, London, SE5 9RS UK; 2 King’s College London, London, WC2R 2LS UK; 3 Institute of Liver Studies, King’s College Hospital, London, UK; 4 Biosciences Institute, Faculty of Medical Sciences, Newcastle University, Central Parkway, Newcastle Upon Tyne, NE1 3BZ UK; 5 The Newcastle upon Tyne Hospitals NHS Foundation Trust, Renal Services, Freeman Road, Newcastle Upon Tyne, NE7 7DN UK; 6 NIHR Newcastle Biomedical Research Centre, Newcastle University, Newcastle Upon Tyne, NE4 5PL UK

**Keywords:** Ciliopathy, Paediatrics, Liver, Molecular genetics, Congenital hepatic fibrosis, Ductal plate malformation, Polycystic liver disease, Genetic testing

## Abstract

Ciliopathies are genetically heterogeneous disorders of primary cilia that frequently involve the liver in childhood, producing a spectrum of hepatobiliary pathology. Primary cilia on cholangiocytes and hepatic progenitors are mechano-, chemo-, and osmo-sensors essential for remodelling of the ductal plate, the embryologic precursor of the liver’s intrahepatic bile duct system. Disruption of the components of primary cilia (basal body, intraflagellar transport (IFT), transition-zone, trafficking, and endoplasmic reticulum (ER)/Golgi protein homeostasis components) leads to ductal plate malformation, aberrant biliary branching, and progressive periductal fibrosis. Classical loci (*PKHD1*, *PKD1, PKD2*) more commonly associated with cystic kidney disease remain central to aetiology, while ER/glycosylation protein homeostasis genes (*GANAB*, *ALG8*, *PRKCSH*, *SEC63*, *DNAJB11*), trafficking/IFT-A and WDR/dynein-2 components (*TULP3*, WDR family) are increasingly recognised. Liver disease most often appears from childhood to adolescence in autosomal recessive polycystic kidney disease (ARPKD), Bardet–Biedl syndrome, Joubert syndrome, and WDR-related disorders, while autosomal dominant polycystic kidney and liver disease (ADPKD and ADPLD) usually presents in adulthood. The main disease patterns also vary: congenital hepatic fibrosis and portal hypertension in ARPKD, hepatic cysts in ADPKD, steatotic liver disease in Bardet–Biedl syndrome, and transaminitis or fibrocystic liver disease in Joubert and WDR-related disorders. Emerging contributors such as *DCDC2* and *PKD1L1* implicate microtubule–cilium interactions and mechanosensory dysfunction in neonatal cholestasis and biliary atresia. Diagnosis increasingly relies on broad multigene panels or exome/genome sequencing, with functional assays guiding interpretation of novel alleles. *Conclusion*: This narrative review synthesises contemporary knowledge on ciliary roles in biliary development, the clinical hepatic phenotypes observed in children and adults, and the evolving genetic landscape with emphasis on discoveries from recent sequencing cohorts and functional studies.

**Table:** 

**What is Known:** • *Ciliopathies are genetically heterogeneous disorders in which primary cilia dysfunction disrupts cholangiocyte signalling and ductal plate remodelling, leading to a spectrum of paediatric hepatobiliary disease ranging from congenital hepatic fibrosis and portal hypertension to cystic liver disease, steatotic liver disease, and neonatal cholestasis*.	
**What is New:** • *Recent genomic and functional studies have broadened the spectrum of ciliopathy-associated genes implicated in paediatric hepatic disease. Clinicians caring for these children should maintain a high index of suspicion as the range of reported phenotypes continues to expand*.

**What is Known:**

• *Ciliopathies are genetically heterogeneous disorders in which primary cilia dysfunction disrupts cholangiocyte signalling and ductal plate remodelling, leading to a spectrum of paediatric hepatobiliary disease ranging from congenital hepatic fibrosis and portal hypertension to cystic liver disease, steatotic liver disease, and neonatal cholestasis*.

**What is New:**

• *Recent genomic and functional studies have broadened the spectrum of ciliopathy-associated genes implicated in paediatric hepatic disease. Clinicians caring for these children should maintain a high index of suspicion as the range of reported phenotypes continues to expand*.

## Introduction

Ciliopathies constitute a heterogeneous group of inherited disorders caused by dysfunction of primary cilia, with broad effects on organogenesis and tissue homeostasis. In children, hepatobiliary manifestations include cystic liver disease, ductal plate malformation (DPM), congenital hepatic fibrosis (CHF), and cholangiopathy as components of multisystem ciliopathies and influence morbidity and long-term outcomes. Recent progress in sequencing technologies and international cohort studies have substantially extended the catalogue of genes associated with hepatic phenotypes, including genes traditionally linked to polycystic kidney and liver disease that also operate via ciliary or cilia-related pathways [1, 2]. Recent evidence implicates multiple WD repeat (WDR) proteins, components of intraflagellar transport (IFT), and dynein-2 complexes, in syndromic ciliopathies with hepatic fibrocystic disease [3, 4]. This narrative review focuses on genetic aetiologies of paediatric ciliopathy-associated liver disease, integrating recent discoveries, genotype–phenotype relationships, and implications for diagnostic evaluation.

## Biology

**Fig. 1:**
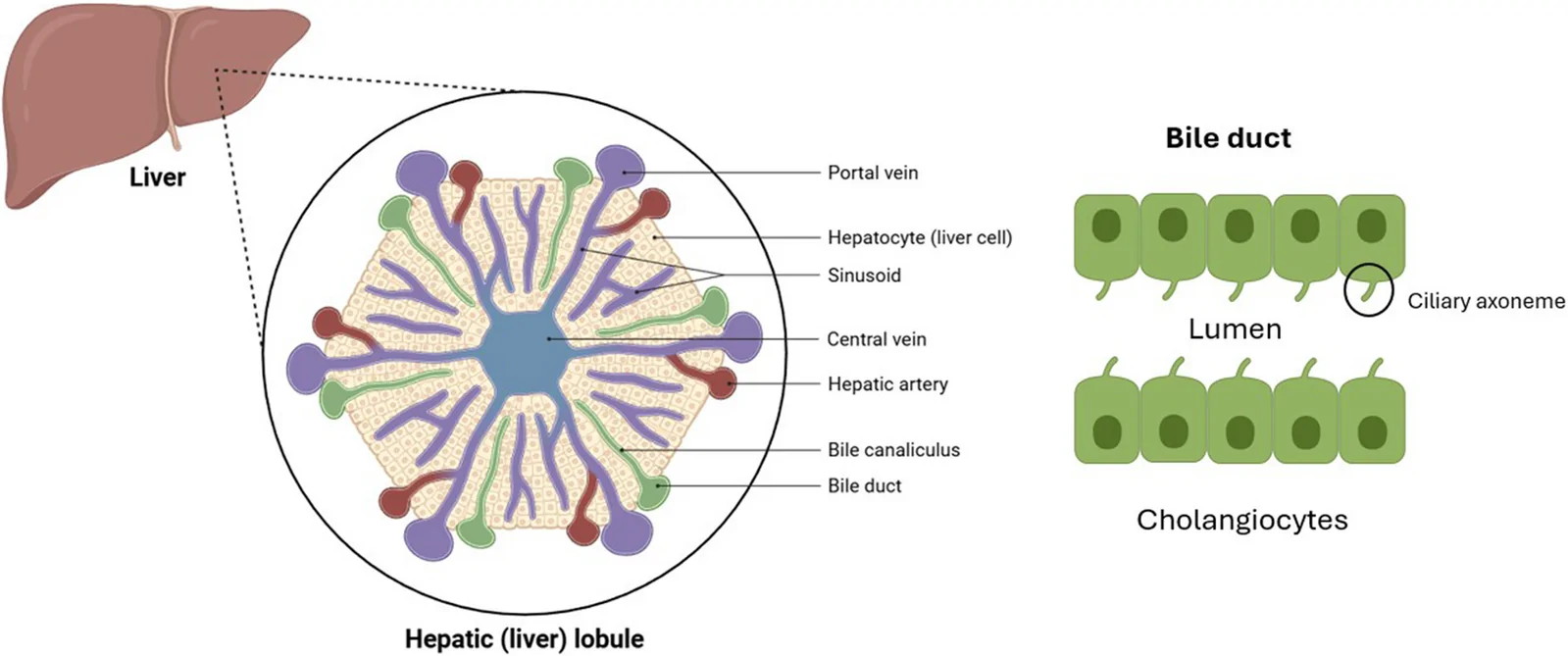
The microscopic organisation of the liver including cholangiocytes and ciliary axoneme. The microscopic organisation of the liver consists of the portal triad (portal vein, hepatic artery, and bile duct) and central vein. Bile ducts are lined with cholangiocytes with primary cilia extending from the cholangiocyte apical plasma membrane into the ductal lumen. These primary cilia have mechano-, osmo-, and chemo- sensory properties regulating bile formation and flow. Created in https://BioRender.com

**Fig. 2:**
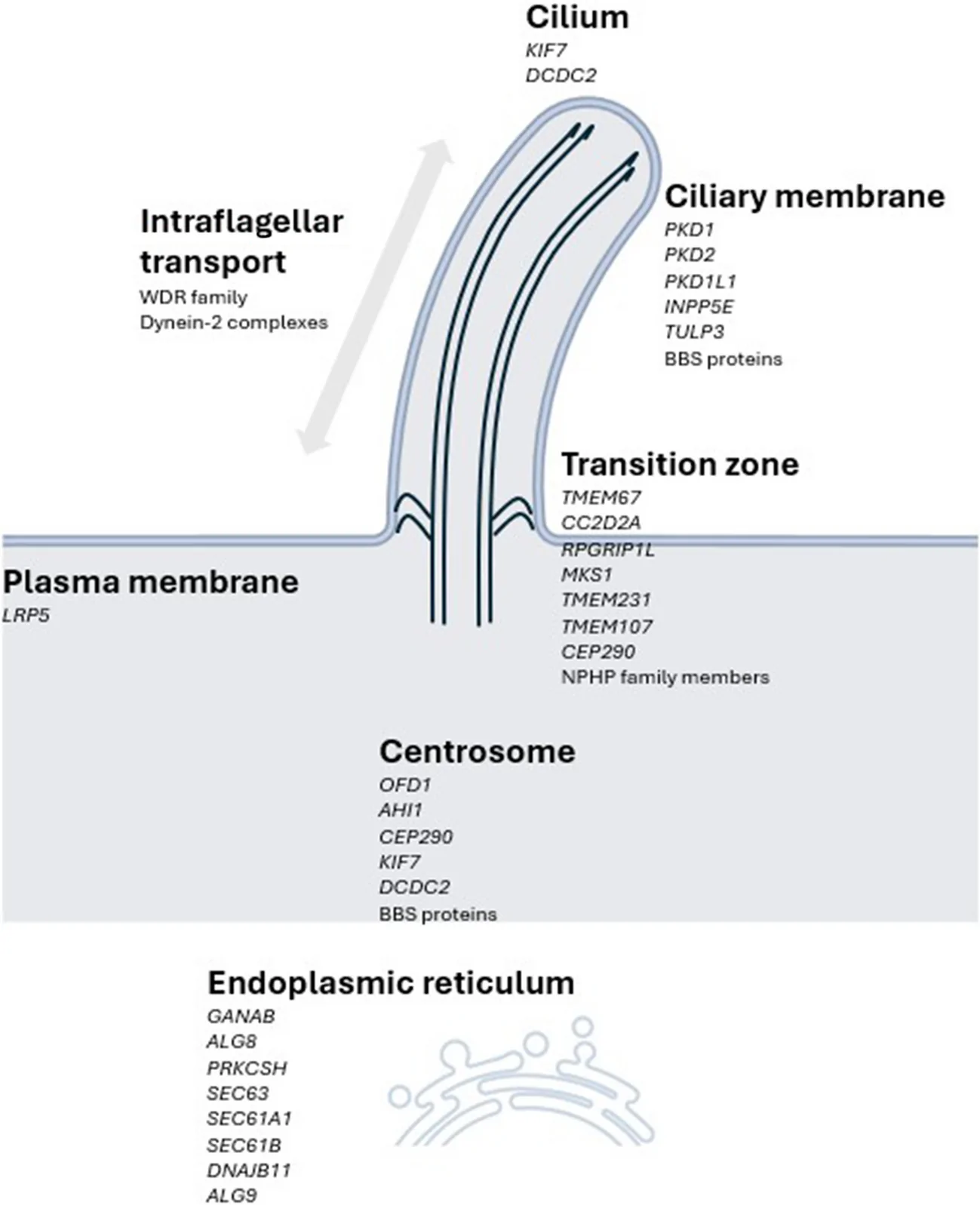
Paediatric hepatic ciliopathies: simplified demonstration of associated proteins and their cellular localisation. Proteins associated with hepatic ciliopathies and their primary subcellular localisation. Ciliary proteins are trafficked from the endoplasmic reticulum/golgi to the ciliary base. The centrosome is the primary microtubule organising centre required for cilia formation [47]. The transition zone separates the cytosol to the nascent cilium functioning as gate to control ciliary composition [5]. Intraflagellar transport mediates the antegrade and retrograde trafficking of proteins. The ciliary membrane and plasma membrane are contiguous expressing proteins regulating mechanosensory and signalling pathways. Created in https://BioRender.com

Primary cilia on cholangiocytes and hepatic progenitors act as sensory and signalling hubs, mediating pathways essential for bile duct morphogenesis and biliary homeostasis (Hedgehog, Wnt, platelet-derived growth factor receptor (PDGFR), transforming growth factor-beta (TGF-β) signalling interplay) [5]. Ciliary proteins, basal body components, IFT complexes, transition zone proteins, and ciliary trafficking adaptors coordinate ductal plate remodelling during embryogenesis. Disruption of these components perturbs cholangiocyte polarity, proliferation, and interaction with mesenchymal cells, promoting DPM, aberrant biliary branching, and periductal fibrosis. Recent mechanistic studies emphasise ciliary trafficking (TULP3-mediated G protein-coupled receptor (GPCR) and signalling cargo transport), IFT integrity (IFT-A/IFT-B and dynein-2 complexes involving WDR proteins), and ER/Golgi processing of membrane and secreted proteins (*ALG8*, *GANAB*, *PRKCSH*) that indirectly affect ciliary function or cholangiocyte biology [6–8]. Defects in WDR proteins compromise retrograde IFT and dynein-2 function, altering cholangiocyte ciliary signalling and contributing to DPM in syndromic presentations [9]. DCDC2 and microtubule-associated factors influence ciliary stability and cholangiocyte architecture, further linking cytoskeletal dynamics to biliary development [10] (Figs. 1 and 2).

## Hepatic manifestations of ciliopathies

**Fig. 3:**
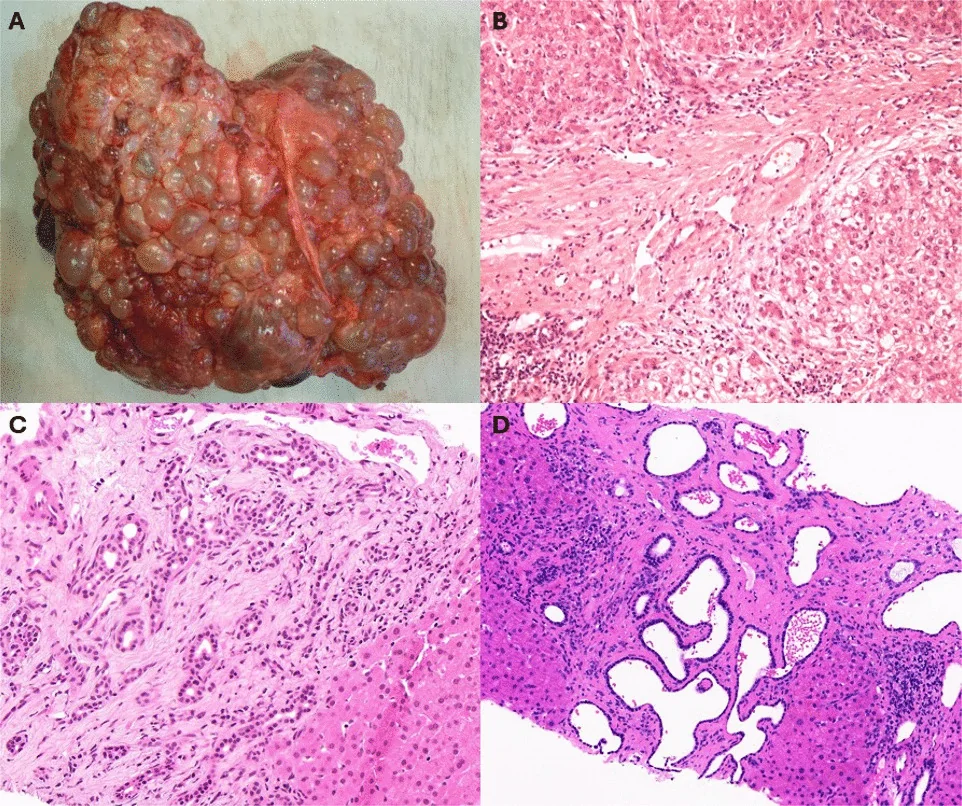
Liver histological features of ciliopathies. **A** Polycystic liver—the liver is enlarged with numerous cysts. **B** Biliary cirrhosis with bile duct loss; no bile ducts are observed in the fibrous septa. **C** Congenital hepatic fibrosis—the parenchyma is divided by fibrous septa containing many small ducts. **D** Ductal plate malformation—the portal tract contains many dilated ducts, some showing irregular papillary protrusion, in keeping with ductal plate malformation

Ciliopathies are clinically heterogeneous, reflecting the ubiquity of primary cilia in organogenesis and tissue homeostasis (Fig. 3). In children, presentations range from multisystem developmental syndromes manifesting at birth to organ-predominant phenotypes that emerge in infancy, childhood or adolescence. Hepatic involvement is a common and clinically important component of many ciliopathies, ranging from developmental DPM to progressive fibrotic and cystic disease. Phenotypes span CHF with portal hypertension, biliary dysgenesis and paucity, neonatal or infantile cholestasis, isolated or predominant polycystic liver disease (PLD) and steatosis; patterns and severity vary by genotype, allelic class and age at presentation (Table 1).

**Table 1:** Hepatic manifestations of ciliopathies—presenting age and features of those encountered in paediatrics

Condition	Typical presenting age (liver disease)	Predominant hepatic phenotype
ARPKD (*PKHD1*)	Childhood—young adults	Congenital hepatic fibrosis and portal hypertension; variable severity by allele
ADPKD (*PKD1*/*PKD2*)	Adulthood; childhood–adolescence in biallelic or digenic variants	Hepatic cysts common in adults; marked paediatric liver disease is uncommon but occurs in early-onset cases
Bardet–Biedl syndrome	Childhood—adolescence	Metabolic steatosis/NAFLD often apparent in childhood/adolescence associated with obesity and insulin resistance
Joubert syndrome	Childhood—adolescence	Transaminitis to fibrocystic liver disease when hepatic involvement present; timing depends on specific gene/alleles
WDR family	Neonate—childhood	Syndromic presentations often include early hepatic involvement within broader disease
*TULP3*	Childhood—adolescence	Fibrocystic liver disease typically presents in childhood but is variable
*DCDC2*	Neonate—childhood	Associated with neonatal sclerosing cholangitis; fibrocystic liver disease
*GANAB */ *ALG8 */ *PRKCSH */ *SEC63 */ *DNAJB11*	Adolescence—adulthood; occasional paediatric cases	Typically polycystic liver disease but can also manifest as congenital hepatic fibrosis and portal hypertension

### Ductal plate malformation and hepatic fibrosis

Developmental ductal plate malformation and CHF are the prototypic hepatic lesion arising from disrupted cholangiocyte ciliary signalling during bile duct morphogenesis. Histologically, this manifests as persistence and disorganisation of embryonic ductal plate remnants and progressive periductal fibrosis, collectively described as CHF (Fig. 3). Clinically, CHF typically presents in infancy or childhood with hepatosplenomegaly, hypersplenism (leading to thrombocytopenia), progressive portal hypertension, and its complications. Congenital hepatic fibrosis is classically associated with autosomal recessive polycystic kidney disease (ARPKD). On abdominal ultrasonographic imaging, liver architecture shows coarse echotexture, biliary dilatation, and associated splenomegaly; magnetic resonance cholangiography can demonstrate fibrotic bands and biliary branching defects and signs of portal hypertension including splenomegaly and vascular collaterals.

### Biliary dysgenesis, cholestasis, and neonatal presentations

Some ciliopathies present in the neonatal or infant period with cholestasis due to biliary hypoplasia, ductopenia, or severe ductal plate disruption. Neonatal cholestasis may be the first manifestation prompting evaluation; distinguishing ciliary-related DPM from other causes (metabolic, infectious, immune) requires attention to concomitant renal cystic disease, syndromic features or suggestive neuroimaging. Progression of cholestasis varies: some infants stabilise with supportive care, whereas others develop early portal hypertension and progressive fibrosis.

### Cystic liver disease and polycystic phenotypes

Genes more traditionally implicated in PLD and atypical ADPKD phenotypes (*PKD1*, *PKD2*) and those involved in ER glycosylation/protein folding (*GANAB*, *ALG8*, *PRKCSH*, *SEC63*, and *DNAJB11*) often present with predominant cystic change. In paediatric cohorts, cystic liver disease may be detected incidentally on ultrasound or as part of familial screening. However, cases with substantial cyst burden and early symptoms have been reported. Cystic disease typically manifests as multiple biliary-derived cysts within an otherwise non-cirrhotic liver, with symptoms related to mass effect, obstructive jaundice, pain or complications such as cholangitis.

### Metabolic liver disease and steatosis

Increasing evidence links certain ciliopathies, most notably Bardet–Biedl syndrome and other syndromes with obesity and metabolic dysfunction, to early-onset hepatic steatosis. In these cases, fatty infiltration and metabolic injury rather than DPM drive hepatic pathology; risk correlates with obesity, insulin resistance, and dyslipidaemia.

### Syndromic context and multisystem correlations

Hepatic manifestations rarely occur in isolation in paediatric ciliopathies. Congenital hepatic fibrosis and biliary anomalies commonly coexist with renal cystic disease, retinal dystrophy, skeletal dysplasia, or neurodevelopmental features. Conversely, PLD associated with ER/glycosylation genes may present predominantly with hepatic cysts and milder extrahepatic findings. WDR and IFT gene defects often produce syndromes that combine skeletal, renal, and hepatic involvement; *DCDC2* and *TULP3* variants have emerged as causes of fibrocystic liver disease within broader ciliopathy spectra [10].

### Links to biliary atresia

Emerging data implicate ciliopathies, specifically *PKD1L1* in the pathogenesis of some biliary atresia cases. *PKD1L1* encodes a ciliary membrane protein involved in mechanosensation and calcium signalling within cholangiocyte cilia. Loss or dysfunction can lead to organ laterality defects and impair bile-flow sensing, cholangiocyte polarity, and ductal plate remodelling. Case reports and experimental models link *PKD1L1* variants to biliary obliteration, phenocopying many features of syndromic biliary atresia with differential expressions of genes mediating ciliary signalling [11–13]. Potentially deleterious biallelic variants were identified in 5 (7%) subjects amongst a cohort of 67 with biliary atresia and splenic malformation syndrome [12]. Broader ciliogenesis and planar polarity effects genes were implicated in a genome-wide association study of 100 biliary atresia cases [14]. These observations support considering *PKD1L1* and other ciliary genes in genetic evaluation of atypical or syndromic biliary atresia and call for further mechanistic studies targeting ciliary signalling.

### Natural history and variability

The hepatic course is highly variable: CHF often leads to childhood portal hypertension but may permit long periods of compensated liver function; cystic disease usually progresses slowly but can be symptomatic early in some genotypes. Age at onset, progression rate, and complications depend on genotype, specific variants (null versus hypomorphic), and modifying factors such as metabolic status as well as female sex [15]. Intrafamilial variability is common [16].

### Hepatic ciliopathies encountered in adults

In adults, ciliopathy‑related liver disease most commonly presents as PLD or as late manifestations of childhood fibrocystic disorders Pathogenic variants in ADPKD genes (*PKD1*, *PKD2*) and in isolated PLD genes (*GANAB*, *PRKCSH*, *SEC63*, *ALG8*, *ALG9*, *DNAJB11*) underlie most adult PLD phenotypes. The overall prevalence of hepatic cysts, amongst 230 15- to 46 year-olds with ADPKD, was 83% in comparison to 58% amongst 15- to 24 year-olds [17]. Adult presentations include progressive cyst burden causing hepatomegaly, abdominal discomfort, early satiety, and complications such as cyst infection, haemorrhage, or biliary compression. Less commonly, adults may manifest consequences of CHF including portal hypertension, splenomegaly, and variceal bleeding, either from surviving ARPKD or from milder, hypomorphic alleles in *TMEM67*, NPHP‑related genes, or *WDR/IFT* loci. The incidence of ARPKD has been estimated as 1 in 20,000 live births; in a cohort of 73 individuals 10 underwent liver transplantation at an average age of 20.3 years [18]. Metabolic liver disease and steatosis also occur in ciliopathy syndromes with obesity/metabolic dysfunction (e.g. Bardet–Biedl syndrome). Pathogenic variants in cilia-related genes may be identified by sequencing analysis of young adults with cryptogenic liver fibrosis [19].

## Molecular genetics

Listed below is an expanded gene-focused catalogue organised by broad functional groups and clinical associations (Table 2). For many genes, hepatic disease occurs as part of a syndromic presentation (multisystem ciliopathy), while others more classically present with isolated or predominant PLD but have mechanistic links to ciliary or cilia-related pathways.

**Table 2:** Genetic causes of hepatic manifestations of ciliopathies

Gene	Protein product	Function	Subcellular location	Primary hepatic phenotype
*PKHD1*	Fibrocystin/polyductin	Receptor-like protein involved in ductal morphogenesis and cell–matrix interactions	Primary cilium; plasma membrane; basal body	Hepatic fibrosis
*TMEM67*	Meckelin	Ciliary transition zone protein; Wnt signalling, ciliogenesis	Transition zone; primary cilium	Hepatic fibrosis
*PKD1*	Polycystin-1	Large transmembrane mechanosensor; forms complex with PC2	Ciliary membrane; plasma membrane	Polycystic liver disease
*PKD2*	Polycystin-2	TRP-like Ca2 + channel; complexes with PC1	Ciliary membrane; ER; plasma membrane	Polycystic liver disease
*GANAB*	Glucosidase II alpha subunit	Catalytic subunit of glucosidase II; ER N-glycan trimming for glycoprotein folding	Endoplasmic reticulum	Polycystic liver disease
*ALG8*	Alpha-1,3-glucosyltransferase	Adds second glucose in N-linked glycosylation; protein folding QC	Endoplasmic reticulum	Polycystic liver disease
*PRKCSH*	Glucosidase II beta subunit	Non-catalytic subunit of glucosidase II; ER proteostasis and glycoprotein maturation	Endoplasmic reticulum	Polycystic liver disease
*SEC63*	SEC63 protein translocation regulator	ER protein translocation/folding with SEC61/SEC62	Endoplasmic reticulum	Polycystic liver disease
*SEC61A1 / SEC61B*	SEC61 alpha 1 subunit	Translocation pore for nascent polypeptides into ER (translocon)	Endoplasmic reticulum/translocon	Polycystic liver disease
*DNAJB11*	DnaJ heat shock protein family member B11	ER co-chaperone of BiP; protein folding/trafficking in ER	Endoplasmic reticulum	Polycystic liver disease
*ALG9*	Alpha-1,2-mannosyltransferase	Adds mannose residues during N-glycan assembly	Endoplasmic reticulum	Polycystic liver disease
*LRP5*	LDL receptor-related protein 5	WNT co-receptor; developmental signalling modulator	Plasma membrane	Polycystic liver disease
*TULP3*	Tub like protein 3	Adaptor for IFT-A-mediated ciliary trafficking (GPCRs/signalling cargos)	IFT-A complex; primary cilium	Hepatic fibrosis
*WDR19*	WD repeat domain 19	Component of retrograde IFT/dynein-2 motor complex	Intraflagellar transport complexes; cilium	Hepatic fibrosis
*DYNC2I2*	Dynein 2 intermediate chain 2	Component of retrograde IFT/dynein-2 motor complex	Intraflagellar transport complexes; cilium	Hepatic fibrosis
*WDR35*	WD repeat domain 35	Component of retrograde IFT/dynein-2 motor complex	Intraflagellar transport complexes; cilium	Hepatic fibrosis
*DYNC2I1*	Dynein 2 intermediate chain 1	Component of retrograde IFT/dynein-2 motor complex	Intraflagellar transport complexes; cilium	Hepatic fibrosis
*RPGRIP1L*	RPGRIP1 like protein	Transitionâ€ ‘zone scaffolding; ciliary gating	Transition zone; basal body	Hepatic fibrosis
*MKS1*	MKS1 protein	Transition zone/basal body scaffold for ciliary gating	Transition zone; basal body	Hepatic fibrosis
*TMEM231*	TMEM231	Transition zone protein	Transition zone; basal body	Hepatic fibrosis
*TMEM107*	TMEM107	Transition zone protein	Transition zone; basal body	Hepatic fibrosis
*TCTN2*	Tectonic family member 2	Transition zone protein	Transition zone; basal body	Hepatic fibrosis
*B9D1*	B9 domain containing 1	Transition zone protein	Transition zone; basal body	Hepatic fibrosis
*CC2D2A*	CC2D2A protein	Transition zone/centrosomal role in ciliogenesis	Transition zone; basal body	Hepatic fibrosis
*CEP290*	Centrosomal protein 290	Transition zone/ciliary gating and trafficking	Transition zone; centrosome	Hepatic fibrosis
*AHI1*	Jouberin	Ciliary signalling scaffolding (Joubert gene)	Cilium; centrosome	Hepatic fibrosis
*INPP5E*	INPP5E	Ciliary membrane phosphoinositide regulator	Ciliary membrane	Hepatic fibrosis
*KIF7*	Kinesin family member 7	Ciliary tip kinesin; Hedgehog pathway regulator	Cilium	Hepatic fibrosis
*NPHP1*	Nephrocystin 1	Ciliary/centrosomal protein for tubulogenesis and signalling	Primary cilium; centrosome; transition zone	Hepatic fibrosis
*NPHP3*	Nephrocystin 3	Ciliary/centrosomal protein for tubulogenesis and signalling	Primary cilium; centrosome; transition zone	Hepatic fibrosis
*NPHP4*	Nephrocystin 4	Ciliary/centrosomal protein for tubulogenesis and signalling	Primary cilium; centrosome; transition zone	Hepatic fibrosis
*IQCB1*	IQCB1	Ciliary/centrosomal protein (nephrocystin related)	Primary cilium; centrosome	Hepatic fibrosis
*OFD1*	OFD1 protein	Basal body/centriolar satellite protein required for ciliogenesis	Basal body; centrosome	Hepatic fibrosis
*BBS1*	Bardet-Biedl syndrome 1	Ciliary trafficking complex (BBSome)	Basal body; cilium	Polycystic liver disease (steatosis and fibrosis reported)
*BBS2*	Bardet-Biedl syndrome 1	Ciliary trafficking complex (BBSome)	Basal body; cilium	Polycystic liver disease (steatosis and fibrosis reported)
*DCDC2*	Doublecortin domain containing 2	Microtubule associated factor; cilium stability and architecture	Cilium; cytoskeleton	Hepatic fibrosis
*PKD1L1*	Polycystin like 1	Ciliary membrane mechanosensor involved in bile flow sensing and Ca2 + signalling	Ciliary membrane	Hepatic fibrosis (implicated in biliary atresia/atresia like lesions)

Among the most established causes is *PKHD1*, the principal gene responsible for ARPKD. Considerable allelic heterogeneity contributes to the clinical spectrum. Truncating variants are frequently associated with severe, early-onset renal disease, whereas individuals carrying at least one missense or otherwise hypomorphic allele may survive beyond the neonatal period and develop predominantly or progressively hepatic disease [20, 21]. In one cohort of 78 patients from 68 independent families, CHF was identified in approximately 32%, illustrating the substantial but variable hepatic burden of *PKHD1*-related disease [21]. These observations highlight the importance of considering *PKHD1* even when hepatic disease predominates and renal manifestations are relatively mild.

*PKD1* and *PKD2*, the major genes responsible for ADPKD, are more commonly associated with hepatic cyst formation than with CHF or DPM. Nevertheless, early-onset and overlapping renal–hepatic phenotypes have been described, including in paediatric patients [22]. Polycystin-1 and polycystin-2 proteins form a complex [23] and localise to primary cilia and participate in mechanosensation and intracellular signalling, providing a direct mechanistic connection between polycystin dysfunction, ciliary biology and cystogenesis.

Several other genes implicated in PLD affect the synthesis, folding, maturation, or trafficking of polycystins and other ciliary membrane proteins. Heterozygous variants in *GANAB* can cause relatively mild ADPKD/PLD phenotypes, with *GANAB* encoding the catalytic α-subunit of glucosidase II and contributing to ER glycoprotein folding. Loss of *GANAB* function can impair polycystin protein maturation and ciliary targeting, thereby linking ER protein processing to ciliary dysfunction [24]. Similarly, heterozygous *ALG8* variants have been associated with PLD and renal cystic disease [25]. *ALG8* participates in N-linked glycosylation, and disruption of this pathway may interfere with the maturation of polycystin and other membrane proteins. In contrast, biallelic *ALG8* variants cause ALG8-related congenital disorder of glycosylation, a severe multisystem disorder presenting in infancy or early childhood. Other ER and protein-processing genes, including *PRKCSH*, *SEC63*, *SEC61B*, and *DNAJB11*, have likewise been associated with PLD and atypical ADPKD phenotypes [24]. *DNAJB11* contributes to a distinct autosomal dominant tubulointerstitial kidney disease with cystic features and may modulate polycystin trafficking [26, 27]. In general, these disorders produce hepatic cystic disease rather than the congenital hepatic fibrosis characteristic of *PKHD1* and other classical ciliopathies. *ALG9*, *LRP5*, and *SEC61A1* have also been reported in congenital or adult-onset polycystic liver/kidney phenotypes, although their contribution to paediatric hepatic disease appears less well established [24].

Several other classical ciliopathy genes can produce hepatic fibrosis and biliary abnormalities, particularly when associated with Joubert, Meckel–Gruber, or nephronophthisis-related phenotypes. *TMEM67* is an important example, with variants associated with Meckel–Gruber syndrome, Joubert-related disorders, and nephronophthisis-spectrum disease [28]. Hepatic involvement may include congenital hepatic fibrosis, biliary abnormalities, and progressive portal hypertension. Hypomorphic missense variants can result in substantially milder phenotypes, including progressive cholangiopathy and splenomegaly accompanied by visual impairment and mild cerebellar vermis hypoplasia [29, 30]. In *TMEM67*-related Joubert/Meckel overlap phenotypes, hepatic fibrosis has been reported at increased frequency (OR 17.3, 95% CI 7.2–42.0; *p* < 0.0001), with particularly strong associations between hepatic disease and coloboma (OR 6.5, 95% CI 3.2–13.4) [31]. Other ciliopathy genes, including *CC2D2A*, *CEP290*, *AHI1*, *INPP5E*, and *KIF7*, may also cause hepatic disease, although the frequency and severity of hepatic involvement vary considerably between genotypes [32, 33]. Similarly, nephronophthisis-associated genes such as *NPHP1*, *NPHP3*, *NPHP4*, *IQCB1*, *CEP290*, and *RPGRIP1L* are predominantly associated with renal disease, but particular alleles, especially in *NPHP3*, can occur in syndromic phenotypes involving CHF or biliary dysgenesis [34–37].

The Meckel–Gruber syndrome phenotypic spectrum further illustrates the overlap between severe developmental ciliopathies and hepatic disease. Genes including *MKS1*, *TMEM67*, *CEP290*, *CC2D2A*, *TCTN2*, *B9D1*, and *B9D2* are associated with severe early-onset phenotypes in which DPM, biliary dysgenesis and hepatic fibrosis may accompany neurological, renal, ocular and skeletal abnormalities. The severity of hepatic disease is again influenced by residual gene function: biallelic loss-of-function variants are more likely to result in severe neonatal or multisystem disease, whereas hypomorphic combinations may permit survival with progressive hepatic fibrosis or a more organ-predominant phenotype [38, 39].

Several additional ciliopathy-associated disorders may include hepatic disease, although the relationship with DPM and CHF is less specific. Bardet–Biedl syndrome, caused by biallelic variants in *BBS* genes (*BBS1–BBS21*), is characterised predominantly by retinal dystrophy, obesity, polydactyly, and renal abnormalities. Hepatic steatosis and metabolic liver disease are increasingly recognised, particularly in association with obesity and metabolic dysfunction, although this phenotype differs mechanistically from the congenital biliary fibrosis seen in classical DPM-associated ciliopathies [31]. *OFD1* can also be associated with hepatic fibrosis in severe syndromic presentations. Likewise, biallelic *DCDC2* variants have been linked to fibrocystic liver disease with renal involvement, with reported phenotypes ranging from cholestatic disease to ductal plate abnormalities and with severity influenced by the underlying alleles.

Increasing evidence also implicates genes involved directly in ciliary trafficking and IFT in paediatric fibrocystic liver disease. *TULP3*, which mediates the trafficking of selected G-protein-coupled receptors and other signalling proteins into primary cilia through the IFT-A complex, has recently been associated with fibrocystic liver disease and biliary abnormalities in children and young adults [2, 40]. Some affected individuals subsequently develop hypertrophic cardiomyopathy, emphasising the multisystem nature of these disorders. Disruption of TULP3-dependent trafficking can alter ciliary signalling and downstream pathways involved in biliary development and fibrosis.

Similarly, defects in IFT-A and dynein-2 components, including WDR proteins such as *WDR19* and *WDR35* and genes encoding dynein-2 intermediate chains, can produce severe ciliopathies with variable hepatic involvement [3, 41, 42]. These disorders encompass Jeune asphyxiating thoracic dystrophy, short-rib polydactyly syndromes and Meckel–Gruber-like phenotypes, and may include hepatic fibrosis or biliary dysgenesis alongside skeletal, renal, retinal, dental and neurodevelopmental abnormalities. Impaired retrograde IFT is likely to disrupt ciliary signalling in cholangiocytes and interfere with normal duct development. The severity of hepatic and systemic disease may also depend on residual protein function, with individuals carrying two missense variants sometimes showing a milder phenotype than those with biallelic frameshift variants [2, 3].

Other transition-zone and ciliary-gating genes, including *RPGRIP1L*, *MKS1*, *TMEM231*, *TMEM107*, and *TMEM67*, can similarly produce a continuum from severe neonatal ciliopathy to later-onset hepatic fibrosis [38, 39]. These proteins contribute to the organisation of the ciliary transition zone and the selective distribution of signalling proteins within the cilium. Their disruption can therefore affect multiple developmental signalling pathways simultaneously, explaining the combination of biliary, renal, neurological, ocular and skeletal manifestations observed in affected individuals.

## Recent advances and translational insights

The use of large genomic biobank studies has led to the discovery of novel genetic causes of ciliopathies where previously in traditional studies milder or asymptomatic patients may be underrepresented. Consequently, disease panels have expanded and diagnostic yield increased revealing unexpected genes (*GANAB*, *ALG8*, *TULP3*, *DNAJB11*, WDR family genes and *DCDC2*) in paediatric cohorts.

Furthermore, in vitro work on *TULP3*, IFT-A components and WDR proteins clarified mechanisms of ciliary trafficking and retrograde IFT critical for cholangiocyte signalling and ductal plate remodelling [43–45]; *DCDC2* studies implicate microtubule associated proteins to interfere with tubulin and eventual development of cholangiocyte architecture [10].

Functional studies demonstrate that ER proteostasis can be linked to ciliopathy phenotypes such as the ER glycosylation defects (*GANAB*, *ALG8*) that impair polycystin maturation and ciliary targeting [4, 7, 24].

Patient-derived organoids and animal models (mouse, zebrafish) investigating biliary atresia have enabled testing of allele-specific effects on biliary morphogenesis, establishing platforms for future mechanistic and therapeutic research [11, 46].

### Key clinical insights from a hepatologist’s perspective

In a patient with otherwise unexplained liver disease, a ciliopathy should be considered in the differential diagnosis especially when imaging shows biliary dilation, hepatic/renal cysts, cholangiopathy and/or portal hypertension; liver biopsy shows DPM and/or biliary cirrhosis. When a primary ciliopathy is the underlying diagnosis, the dominant hepatic phenotype may be either: DPM leading to CHF or fibrocystic liver disease both leading to progressive portal hypertension. Portal hypertension can vary from being clinically silent with subclinical splenomegaly and mild thrombocytopenia, to clinically significant with varices, ascites, and hypersplenism. Clinical risk stratification should combine genotype (if available), biochemical indices of liver function, radiological findings of portal hypertension and non-invasive portal-hypertension markers (such as platelet count or elastography) with endoscopic surveillance reserved for those with evidence of clinically significant portal hypertension. Ultimately, liver transplantation is indicated for end-stage liver disease considering the trajectory of the renal disease.

## Conclusions

Recent genetic advances have expanded the spectrum of genes implicated in paediatric hepatobiliary disease within the ciliopathy continuum. Beyond classical ciliopathy loci, ER/glycosylation and proteostasis genes, trafficking regulators, IFT-A/dynein-2 and WDR components and microtubule/ciliary dynamics genes are increasingly recognised disease contributors. Comprehensive genetic evaluation, using broad panels or exome/genome sequencing with careful variant interpretation, is essential to clarify diagnosis, anticipate organ involvement and prioritise research. Continued genotype–phenotype mapping and functional validation will be critical to understand mechanisms linking diverse genes to hepatic pathology in children.

## Data Availability

No datasets were generated or analysed during the current study.

## Abbreviations

ADPKD: Autosomal recessive polycystic kidney disease
ARPKD: Autosomal recessive polycystic kidney disease
CHF: Congenital hepatic fibrosis
DPM: Ductal plate malformation
ER: Endoplasmic reticulum
IFT: Intraflagellar transport
PDGFR: Platelet-derived growth factor receptor
PLD: Polycystic liver disease
TGF-β: Transforming growth factor-beta
WDR: WD repeat
